# Low-Dose Ketone Monoester Administration in Adults with Cystic Fibrosis: A Pilot and Feasibility Study

**DOI:** 10.3390/nu16223957

**Published:** 2024-11-19

**Authors:** Eric P. Plaisance, Jonathan M. Bergeron, Mickey L. Bolyard, Heather Y. Hathorne, Christina M. Graziano, Anastasia Hartzes, Kristopher R. Genschmer, Jessica A. Alvarez, Amy M. Goss, Amit Gaggar, Kevin R. Fontaine

**Affiliations:** 1 Department of Nutrition Sciences, University of Alabama at Birmingham, Birmingham, AL 35294, USA; 2 Division of Pulmonary, Allergy, & Critical Care Medicine, University of Alabama at Birmingham, Birmingham, AL 35294, USAkristophergenschmer@uabmc.edu (K.R.G.);; 3 Department of Health & Kinesiology, University of Utah, Salt Lake City, UT 84112, USA; 4 Department of Human Studies, University of Alabama at Birmingham, Birmingham, AL 35294, USA; graziano@uab.edu; 5 Department of Biostatistics, University of Alabama at Birmingham, Birmingham, AL 35294, USA; 6 Division of Endocrinology, Metabolism and Lipids, Department of Medicine, Emory University, Atlanta, GA 30322, USA; jessica.alvarez@emory.edu; 7 Department of Health Behavior, University of Alabama at Birmingham, Birmingham, AL 35294, USA; kfountai1@uab.edu

**Keywords:** ketones, ketogenic diet, low-carbohydrate diet, pulmonary function, inflammation

## Abstract

Introduction: Cystic fibrosis transmembrane conductance regulator (*CFTR*) modulators have greatly improved outcomes in persons with CF (pwCF); however, there is still significant heterogeneity in clinical responses, particularly with regard to respiratory infection and inflammation. Exogenous administration of ketones has profound systemic anti-inflammatory effects and produces several nutrient-signaling and metabolic effects that may benefit multiple organ systems affected in pwCF. This pilot study was designed to determine the feasibility of administration of a ketone monoester (KME) to increase circulating D-beta hydroxybutyrate concentrations (D-βHB) and to improve subjective measures of CF-specific quality of life and markers of inflammation in serum and sputum in adults with CF. Methods: Fourteen participants receiving modulator therapy were randomized to receive either KME (n = 9) or placebo control (PC, n = 5) for 5–7 days during hospitalization for treatment of acute pulmonary exacerbation or as outpatients under standard care. Results: The KME was well tolerated, with only mild reports of gastrointestinal distress. D-βHB concentrations increased from 0.2 ± 0.1 mM to 1.6 ± 0.6 mM in the KME group compared to 0.2 ± 0.0 to 0.3 ± 0.1 in the PC group (*p* = 0.011) within 15 min following consumption and remained elevated, relative to baseline, for over 2 h. Pulmonary function was not altered after single- or short-term KME administration, but participants in the KME group self-reported higher subjective respiratory scores compared to PC in both cases (*p* = 0.031). Plasma inflammatory markers were not statistically different between groups following the short-term (5–7 d) intervention (*p* > 0.05). However, an exploratory analysis of plasma pre- and post-IL-6 concentrations was significant (*p* = 0.028) in the KME group but not PC. Sputum IFNγ (*p* = 0.057), IL-12p70 (*p* = 0.057), IL-1β (*p* = 0.100), IL-15 (*p* = 0.057), IL-1α (*p* = 0.114), and MPO (*p* = 0.133) were lower in the KME group compared to PC but did not achieve statistical significance. Conclusions: With the emerging role of exogenous ketones as nutrient signaling molecules and mediators of metabolism, we showed that KME is well tolerated, increases circulating D-βHB concentrations, and produces outcomes that justify the need for large-scale clinical trials to investigate the role of KME on whole-body and tissue lipid accumulation and inflammation in pwCF.

## 1. Introduction

Over 33,000 individuals in the United States have been diagnosed with cystic fibrosis (CF), with over 900 new cases diagnosed each year [1,2,3,4]. Cystic fibrosis is an autosomal recessive genetic variation, resulting from over 700 CF-causing mutations of the cystic fibrosis transmembrane regulator (*CFTR*) gene, with the mutation of phenylalanine 508 accounting for up to 90% of all *CFTR* cases [5,6]. The *CFTR* gene encodes a chloride channel structuring protein that is essential for osmotic balance and maintenance of electrolytes on the apical surface of epithelial cells in the upper and lower respiratory tract, pancreas, liver, gallbladder, intestines, and other exocrine organs [7,8]. Mutation of the gene results in protein misfolding, degradation, and decreased transport and deposition of the *CFTR* protein in the plasma membrane. Decreased transport of chloride and bicarbonate increases viscosity of mucus and intracellular pH, leading to poor gas exchange in the respiratory system, nutrient malabsorption, chronic infection, and inflammation. The long-term consequences of repeated infection and inflammation are the most common causes of respiratory failure and mortality among persons with CF (pwCF) [5].

The NOD-like receptor P3 (NLRP3) inflammasome is a critical component of the innate immune system and is the first line of host defense that mediates caspace-1 activation and secretion of proinflammatory cytokines from macrophages and neutrophils in response to microbial infection and cellular damage [9]. Aberrant activation of the NLRP3 inflammasome has been observed in CF [10]. Rimessi and colleagues showed that the degree and quality of the inflammatory response in CF is supported by *Pseuomonas aeruginosa*-dependent mitochondrial perturbation in the Ca^2+^ uniporter that produces NLRP3 activation and IL-1β and IL-18 secretion [11].

The ketone monoester, R-(3)-hydroxybutyl R-(3)-hydroxybutyrate (KME), has been shown to increase circulating ketone concentrations in a dose-dependent fashion [12,13,14,15] and to inhibit activation of the NLRP3 inflammasome [16]. Youm and colleagues [17] showed that D-beta hydroxybutyrate (D-βHB) decreases activation of the NLRP3 inflammasome independent of succinyl-CoA:3 ketoacid CoA transferase (SCOT) and hydroxycarboxylic acid receptor 2 (HCAR2). Others show that D-βHB is an inhibitor of histone deacetylases (HDACs) and that HDAC inhibition leads to reductions in macrophage IL-6 and IL-12 levels, but not TNFα or MCP-1 through mechanisms that likely include decreased transcription of NF-κB [16,18,19].

The primary purpose of this pilot investigation was to determine whether the exogenous KME is tolerable and whether it increases circulating D-βHB concentrations in pwCF. We also explored whether KME would improve pulmonary function and perceived quality of life and decrease circulating markers of inflammation.

## 2. Materials and Methods

### 2.1. Study Design

This “proof of concept” randomized, double-blind, placebo-controlled pilot and feasibility trial tested the effects of an exogenous KME as an adjunctive therapy on inflammatory markers and selected clinical outcomes in adults diagnosed with CF. Patients were randomly allocated in unbalanced numbers (2:1) [n = 10 KME and n = 5 placebo control (PC)] to receive the KME or a similar-tasting placebo control drink, respectively, for 5–7 days during hospitalization for acute pulmonary exacerbation or as outpatients under standard care.

### 2.2. Participants and Recruitment

Participants were recruited from the inpatient or outpatient CF population within the University of Alabama at Birmingham (UAB) Gregory Fleming James Cystic Fibrosis Research Center. Participants were previously/historically diagnosed with CF using routine genetic testing and/or diagnostic criteria, including sweat Cl^−^ values > 60 mM and a minimum of two clinical features consistent with the diagnosis [20,21]. Inclusion criteria included: diagnosis of CF, age > 19 years, colonization with *Pseudomonas aeruginosa*, acute pulmonary exacerbation (APE) requiring inpatient care, or outpatients receiving standard of care. Exclusion criteria were concurrent or recent (within 28 days of enrollment) use of corticosteroids, acute respiratory failure requiring the use of invasive or noninvasive ventilation, chronic liver or renal disease, and pregnancy. Outpatients were screened based on the most recent prominent eligibility limitation of *Pseudomonas aeruginosa* positive culture reporting within 2 years of screening. Remaining patients who had previously declined were subsequently removed from the outpatient screening list. The study was approved by the UAB Institutional Review Board, with all patients providing written informed consent prior to study participation.

Twenty mL of either KME (KetoneAid, Falls Church, VA, USA; 10.0 g D-βHB per serving) or a comparable visual and tasting PC was administered twice daily for 5–7 days. Treatment occurred following an overnight fast, between 0800 and 1000 h and again at 1500 h for a total of 40 mL (20 g) per day. The KME consists of 49% water, 49% R-(3)-hydroxybutyl, (3)-hydroxybutryate, KME), <2% stevia, citric acid, allulose, natural flavor, and potassium sorbate. The PC was composed of distilled water, stevia, potassium sorbate, natural flavors, and denatonium benzoate.

### 2.3. Randomization and Procedures

**Figure 1:**
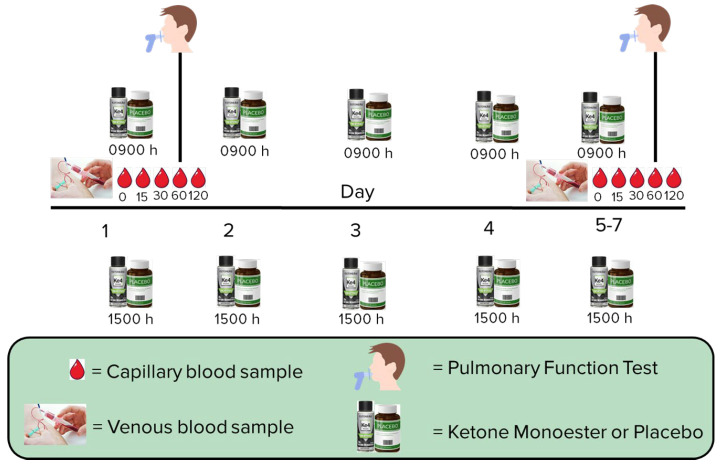
Study flow.

Since this was a single-site, proof-of-concept study, participants were randomized in a 2:1 (KME:PC) fashion in blocks of 4 to improve power and enable better study of the intervention group. On day 1, a fasting baseline blood sample was obtained by venipuncture (Figure 1). A fasting baseline capillary blood sample (T0) was then obtained and D-βHB concentrations were measured using a point of care device. Additional capillary blood samples were obtained at 15, 30, 60, 90, and 120 min. A pulmonary function test (PFT) was obtained on day 1 at T0 and again at 60 min (T60) following the initial dose of KME or PC to examine acute changes in respiratory function. Sputum samples were also obtained at T0 in those participants who were able to spontaneously expectorate sputum. A second dose of KME (10 g) or equivalent volume of PC was provided at approximately 1500 h. On day 5 (outpatient) or days 5–6 (inpatient), a fasting blood sample was obtained by venipuncture to examine the short-term effects of the KME on circulating inflammatory markers. In addition, sputum samples were obtained prior to the final dose of KME or PC (T0) to examine short-term effects of the KME on sputum inflammatory markers. Capillary blood samples were again obtained at 15, 30, 60, 90, and 120 min following the morning dose of KME or PC. A PFT was obtained at T0 and again at T60 following the initial dose of KME or PC on the final day to examine short-term (5–7 d) effects of the KME on respiratory function.

Participants were asked to avoid sugar-sweetened beverages and foods containing added sugar throughout the study to reduce circulating insulin concentrations which have been shown to decrease the magnitude and duration of circulating ketones following an acute dose of KME [13]. Participants remained on all CF chronic care medications and standard inpatient exacerbation therapy (intravenous antibiotics and increased airway clearance) throughout the course of the study as previously described by Xu and colleagues [21].

### 2.4. Study Outcomes

Pulmonary function test (PFT): Spirometry (MGC Diagnostics CPFS, St. Paul, MN, USA) was conducted before (T0) and after 60 min (T60) on day 1 following the first dose of KME or PC and on the final day of the study at T0 and T60; this was to examine the acute and short-term effects of the KME on pulmonary function using American Thoracic Society guidelines [22].

D-βHB and inflammatory markers: Capillary D-βHB concentrations were measured from capillary blood following a finger stick using a lancet and point of care device (KetoMojo, Napa, CA, USA) [15]. Whole blood was obtained by venipuncture. Plasma was isolated from whole blood by centrifugation at 1500× *g* for 10 min and stored at −80 °C until analysis. In patients who were able to produce sputum, samples were collected and processed by adding 10 mL ice-cold PBS to sputum in a 50 mL tube. Sputum was broken up by repeated aspiration through a 20 mL syringe with an 18 G × 1.5-inch needle. Sputum was spun at 800× *g* for 10 min, then the cell-free supernatant was spun again at 3000× *g* for 10 min. Debris free sputum supernatant was then aliquoted and frozen at −80 °C until use. Inflammatory markers thought to be relevant to CF (e.g., IL-6, IL-10, IL-18, TNFα) were measured in serum and sputum by the UAB Metabolism Core of the Nutrition Obesity Research Center using instructions provided by the manufacturer.

Study questionnaires: Cystic Fibrosis Questionnaire—Revised Adolescent/Adult (CFQ-R) is a validated instrument for adolescents and adults (≥14 years of age), widely used to assess quality of life in CF studies and clinical trials [23,24]. The CFQ-R measures daily functioning from the patient’s perspective, and thus provides unique information to facilitate clinical interventions. More broadly, it provides systematic data on the frequency and severity of respiratory symptoms that are not measured using conventional clinical tests [25]. The CFQ-R has 12 dimensions: physical functioning, emotional functioning, social functioning/school functioning, body image, eating problems, treatment burden, respiratory symptoms, digestive symptoms, vitality, health perceptions, weight, and role functioning, with higher scores indicative of better CF-related quality of life. The CFQ-R was administered at approximately 1700 h on day 1 and again on days 5 or 6 to examine effects of the KME or PC on each of the domains assessed by the survey.

Tolerability/Symptom Questionnaire: We assessed symptoms and side effects using a questionnaire that surveilled a series of digestive symptoms (e.g., nausea, heartburn, belching), the timing of the onset of symptoms after consuming the KME or PC, and the duration of the symptoms(s).

### 2.5. Statistical Analysis

Descriptive statistics were calculated for all study measures. The distribution of data was evaluated for normality and non-parametric statistics were employed where the assumption of normality was violated. Comparisons between KME and PC were performed using a 2 group (KME vs. PC) × 2 (baseline vs. follow-up) ANOVA with repeated measures on time. D-βHB concentrations were analyzed using a 2 (group) × 6 (time) repeated measures ANOVA. Wilcoxon Rank–Sum tests were used to explore differences among groups for pulmonary function, survey, and inflammatory marker outcomes. Pre- and post-values were examined in the KME group for exploratory purposes using Wilcoxon Signed Rank testing. Because there were no significant differences on study variables as a function of inpatient vs. outpatient status, the data were combined in the primary analyses. Statistical significance was decided a priori at *p* < 0.05, two-tailed. SAS (version 9.4, SAS Institute, Cary, NC, USA) and SPSS (Chicago, IL, USA, version 28.0.1.1) were used to conduct the analysis.

## 3. Results

### 3.1. Participants

**Figure 2:**
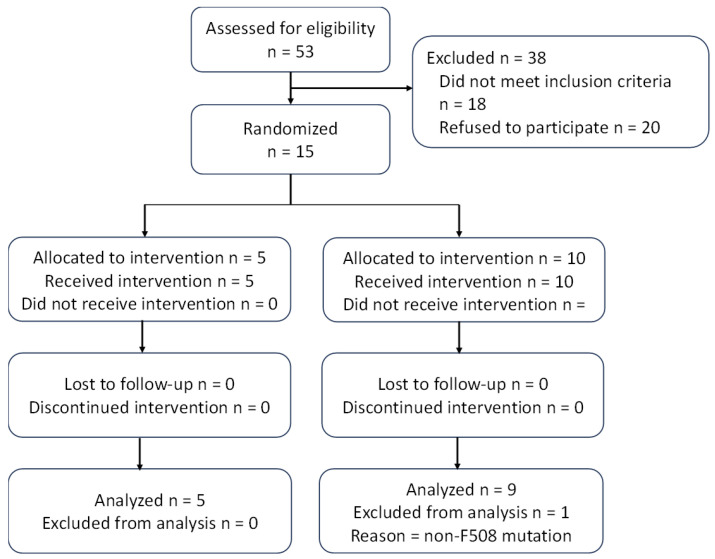
CONSORT diagram.

A total of 15 participants (n = 10 KME and n = 5 PC) were enrolled and 14 were included in the final analysis (Figure 2). All participants were taking elexacaftor/tezacaftor/ivacaftor and approximately 50% had CFRD, accurately representing the adult population (CFRD as documented within medical history and being prescribed insulin therapy) [26]. Out of the 15 participants, 14 had at least a heterozygous mutation for F508, consistent with genetic pwCF representation. The participant that was not heterozygous or homozygous for F508 was removed from the statistical analysis to reduce heterogeneity of the sample. As shown in Table 1, participants ranged in age from 21 to 52 years, with 53% identifying as women, and 93% identifying as European American. Forty percent of participants were admitted for APE, while 60% of participants were outpatients. Table 2 presents the genotypic frequency of the 14 participants and their group assignments.

### 3.2. Effects of the KME on Circulating D-βHB, Acceptability, and Tolerability

**Figure 3:**
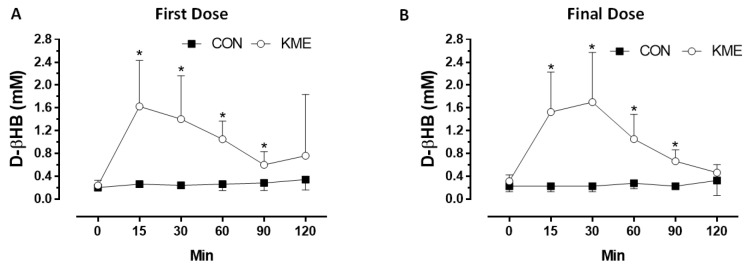
D-beta hydroxybutyrate (D-βHB) after the first dose (**A**) and final dose (**B**) of KME or PC. Values are means ± SD. Significance was set a priori at *p* < 0.05. * = Significant group x time interaction.

Circulating D-βHB concentrations increased rapidly with peak concentration occurring 15–30 min following consumption in the KME group. By 120 min post-consumption, the concentrations remained elevated, but were not significantly different from baseline values (Figure 3). In contrast, there were no significant changes in circulating D-βHB concentrations in the PC group. The KME and PC were well-tolerated, producing only minimal gastrointestinal distress such as gas or bloating that were not different between groups (Table 3).

### 3.3. Pulmonary Function and CFQ-R

Pulmonary function outcomes (FEV_1.0_, FVC, or FEV_1.0_/FVC) were similar between groups following a single dose and 5–7 d after KME or PC. While there were no differences between groups in most CFQ-R domains assessed, self-reported respiratory function was higher in the KME group compared to PC after one day (*p* = 0.0311) but did not meet statistical significance on the final day of treatment (*p* = 0.0544) (Table 4).

### 3.4. Effects of KME on Circulating and Sputum Inflammatory Markers

Overall, there were no statistically significant differences in any of the plasma inflammatory markers measured when comparing differences between groups using Wilcoxon Rank–Sum testing (Table 5). Since this was a pilot and feasibility study with a limited number in the PC group, an exploratory analysis of the KME group differences alone (pre vs. post) using Wilcoxon Signed Rank Testing was conducted and showed that IL-6 [baseline 1.03 (0.20, 3.73 pg/mL) vs. one-week follow-up 0.53 (0.18, 2.08) pg/mL] was significantly lower (z = −2.19, *p* = 0.028) following short-term administration of the KME (no statistically significant difference was observed in the PC group).

**Figure 4:**
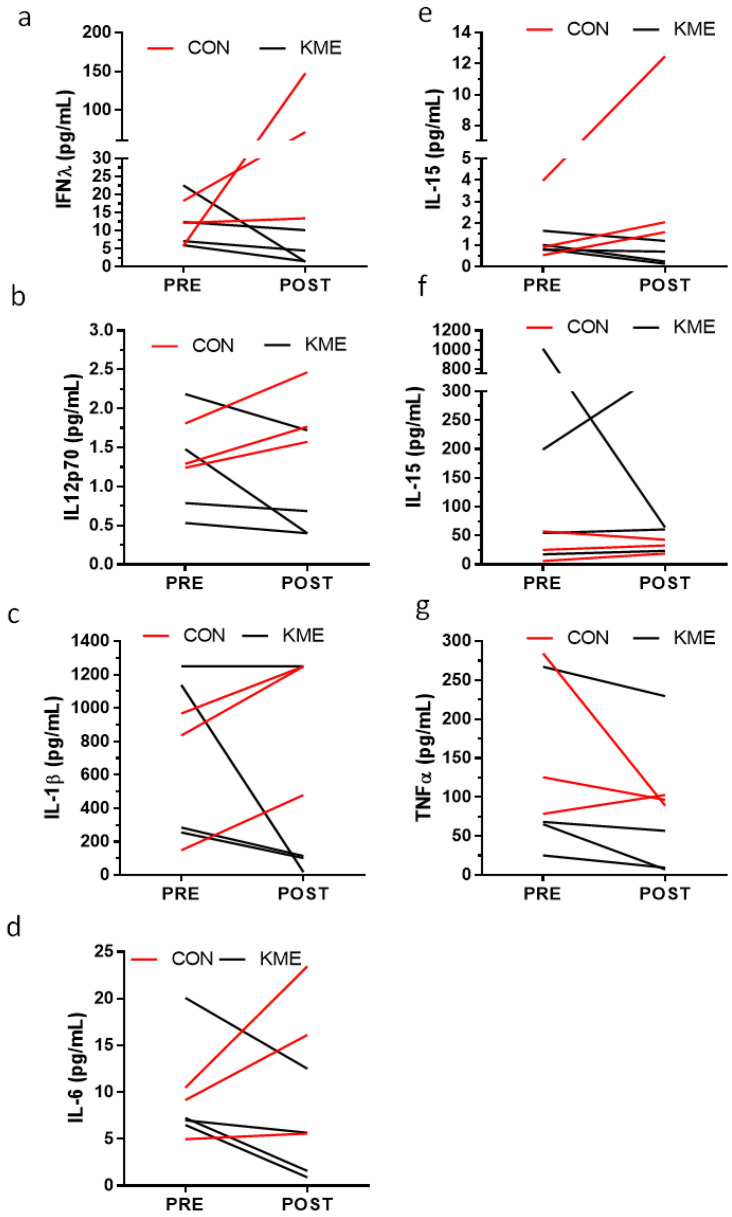
Values are individual sputum inflammatory marker responses for (**a**) interferon γ (IFG), (**b**) interleukin-12p70 (IL-12p70), (**c**) interleukin-1β (IL-1β), (**d**) interleukin 6 (IL-6), (**e**) interleukin 15 (IL-15), (**f**) interleukin 16 (IL-16), (**g**) tumor necrosis factor α (TNFα).

**Figure 5:**
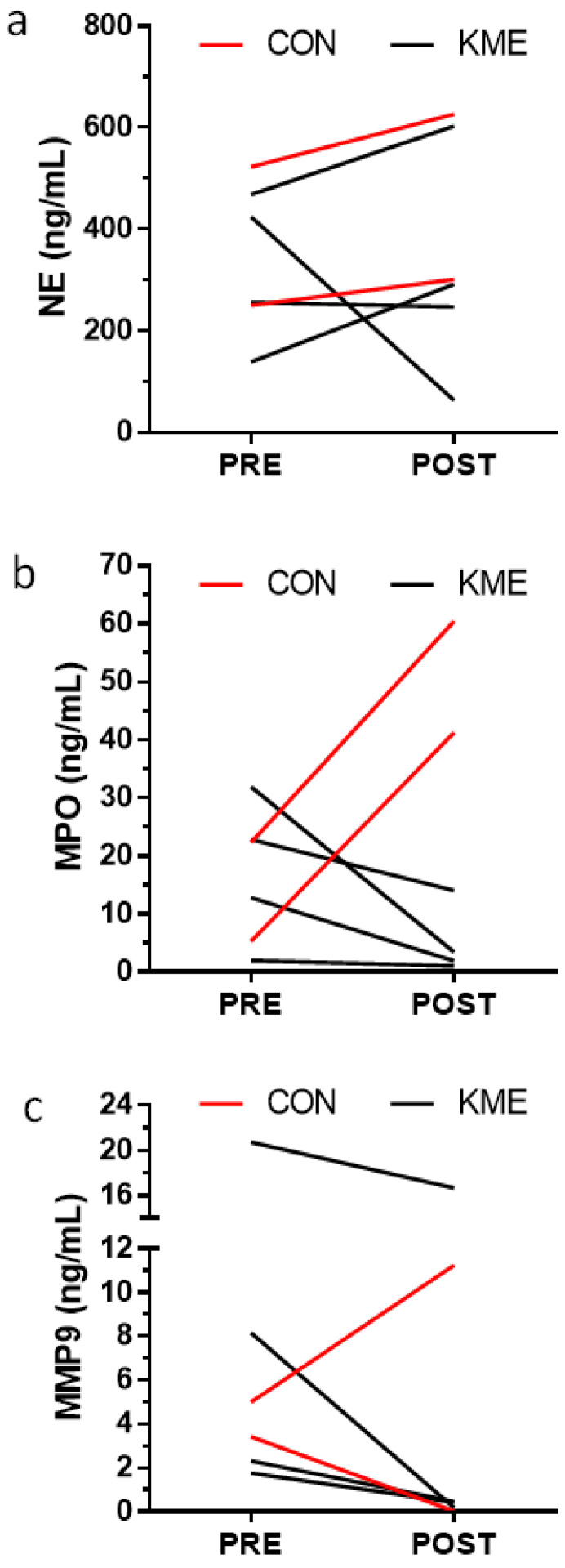
Values are individual sputum inflammatory marker responses. (**a**) NE = neuroelastin, (**b**) MPO = myeloperoxidase, (**c**) MMP9 matrix metalloproteinase 9.

Out of the 9 participants in the KME group, 4 were able to produce sputum before and after the intervention, whereas 3 out of 5 participants in the PC group produced sputum. Despite the small sample size and lack of statistical significance, a comparison of group differences by Wilcoxon Rank–Sum testing is presented in Table 6 and Figure 4 and Figure 5.

## 4. Discussion

To our knowledge, this pilot is the first study to examine the impact of exogenously administered ketones in pwCF. Findings from the current investigation indicate that KME increased circulating D-βHB concentrations at the dosage and frequency administered and was well-tolerated. When the study was conceived and approved for funding, the pivotal highly effective modulator therapy elexacaftor/tezacaftor/ivacaftor had not yet been FDA-approved. It was our initial goal to recruit pwCF who were admitted to UAB Hospital for an APE, but with the dramatic efficacy of the *CFTR* modulators, we soon encountered enrollment challenges that warranted including pwCF who were not admitted for APE. Although including the outpatient cohort would increase the potential variability in the findings, as a pilot and feasibility study, it increased our inferential capacity to most accurately reflect therapeutic intervention in the post-modulator era CF clinical care setting. While the findings suggest that KME had no synergistic or additive to improve pulmonary function in the presence of *CFTR* modulation, participants in the KME group reported a subjective improvement in respiratory function within one day of administration that was not observed in the PC group, as determined by CFQ-R. Furthermore, exploratory analysis of plasma and sputum inflammatory markers suggests that longer-term studies with more participants are justified to examine the effects of KME to reduce inflammation in the presence of CFTR modulation in pwCF.

Increasing circulating ketones through a ketogenic diet, alone or in combination with exogenous ketones, has gained significant interest in the scientific community due to emerging evidence that supports a vast array of improvements in energy homeostasis and metabolism [27,28], skeletal muscle structure and function [29], and systemic inflammation [30]. In this study, one of our key aims was to determine whether administration of the KME at a relatively low dosage would successfully increase circulating D-βHB concentrations in pwCF. Although several studies have demonstrated that this occurs in apparently healthy individuals, there have been few reports examining the kinetics of ketone ester administration in individuals with disease incurring chronic inflammation, including CF. Addressing these limitations is important as we attempt to explore longer-term ketone administration in a number of clinical spaces.

As described previously, kinetic responses to the KME were initially conducted by Clarke and colleagues [12] and demonstrate a dose–response effect in apparently healthy individuals at 140 (~10 g), 357 (~25 g), and 714 (~50 g) mg/kg body weight, producing D-βHB concentrations of 0.28, 1.00, and 3.30 mM, respectively. The Cmax for acetoacetate (AcAc), which is often not measured due to lack of point of care technologies that are available for AcAc, also increased in a dose-dependent fashion, which may also have important implications as a result of the apparent duality of effects produced by D-βHB and AcAc [31,32]. More recent studies in healthy populations provide further confirmation that the KME increases circulating D-βHB in a dose-dependent fashion. Indeed, Stubbs and colleagues [13] showed that administration of the KME at ~12 g increased circulating D-βHB concentrations to approximately 1.5 mM within 15–30 min, that returned to baseline within 2 h following administration. Other studies using the KME at a 10 g dose also increased circulating D-βHB to between 1.6 and 2.4 mM concentrations [15,33]. D-βHB concentrations increased to approximately 1.6 mM in the current study in pwCF after a single dose with the Cmax for the D-βHB occurring within 15 min. Acute assessment after short-term administration (5–7 d) showed only minor kinetic differences with the Cmax occurring at 30 min.

Overall, these findings demonstrate that the KME at the used dose increased circulating D-βHB concentrations successfully and similarly to previous reports in other populations. Future studies are needed to determine whether higher doses are needed to maintain circulating concentrations for longer periods, particularly with the goal of maintaining ketosis throughout the day and over long-term studies. However, these findings at lower doses are promising as the cost of ketones will continue to be a barrier if higher concentrations and frequencies of dosing are needed to maximize benefits.

The KME was generally well-tolerated by the participants, with few reports in the PC and KME groups of gastrointestinal distress. These findings are consistent with those observed in apparently healthy individuals [12]. There were no other reported signs or symptoms associated with either the PC or the KME. It is important to note that all participants but one were prescribed pancrelipase, an oral mixture of lipases, proteases, and amylases for exocrine pancreatic insufficiency, resulting in subsequent nutrient malabsorption. Each of the participants were also prescribed the CFTR modulator, elexacaftor/tezacaftor/ivacaftor, which has been shown to improve nutrient malabsorption. These findings provide evidence that the KME is well tolerated by pwCF, and along with the completion rate of the study, this evidence suggests that long-term administration of the KME is feasible and should be well tolerated.

PwCF present with lower-than-predicted FEV_1.0_ generally because of chronic inflammation and excess mucus production. Multiple RCTs show that the *CFTR* modulators elexacaftor/tezacaftor/ivacaftor increase predicted (*p*) FEV_1.0_ compared to PC [2,34] or with tezacaftor/ivacaftor alone [35]. The administration of KME in the presence of elexacaftor/tezacaftor/ivacaftor did not improve pulmonary function outcomes, suggesting that the KME provides no synergistic or additive effects beyond those produced by the modulators. However, patients receiving the KME reported significantly higher CFQ-R values in the respiratory domain after one day suggesting that subjective feelings of respiratory function were improved despite an absence of functional changes observed with spirometry. The implications of these findings are not clear and warrant further investigation. It is also important to note that not all pwCF are able to take elexacaftor/tezacaftor/ivacaftor due to the genotype, side-effects, or finances.

Although triple combination modulator therapy is dependent upon CFTR mutation class, all participants had a minimum of one copy of the F508del allele. Previous studies demonstrated a 13.6% change in ppFEV1 following four weeks of triple elexcaftor/tezacaftor/ivacaftor administration in clinical trial participants heterozygous for F508del [36]. Any differential response to KME administration among the F508del homozygous and heterozygous cohorts was statistically insignificant and would be reliant upon a myriad of other pathophysiological influences. Furthermore, despite CF being largely recognized as a monogenetic disease, new evidence has highlighted the interplay of modifier genes exhibiting a polygenetic etiology, either preserving membrane gating function [37], or exacerbating a more severe phenotype [38].

A growing body of evidence suggests that D-βHB inhibits activation of the NLRP3 inflammasome and is a moderate inhibitor of certain histone deacetylases (HDACs). Youm and colleagues [17] were among the first to demonstrate that D- and L-βHB decreases activation of the NLRP3 inflammasome in a succinyl-CoA:3 ketoacid CoA transferase (SCOT) and hydroxycarboxylic acid receptor 2 (HCAR2)-independent fashion, suggesting that their potential to reduce inflammation does not require oxidation or HCAR2 receptor signaling. Administration of the HDAC inhibitor, butyrate, has been shown to induce anti-inflammatory effects in group 2 innate lymphoid cells (ILC2s) [39]. In vitro studies in macrophages provide further evidence that butyrate-mediated HDAC inhibition in macrophages reduced IL-6 and IL-12 levels, but not TNFα or MCP-1 levels, through mechanisms that likely include decreased transcription of NF-κB [18,19]. While butyrate appears to be a more powerful inhibitor of HDACs than D-βHB, it seems plausible that the KME/D-βHB would also lead to decreases in inflammatory markers through HDAC inhibition, but this has yet to be tested.

The findings from the present study show that low-dose KME was well-tolerated and increases D-βHB concentrations. In addition, the study provides preliminary data that together justify a large-scale clinical trial in pwCF (particularly for those ineligible for *CFTR* modulator therapy) to determine whether long-term treatment with KME would reduce inflammation and subsequently improve outcomes. Despite the small sample size of this study, we observed biologically relevant reductions in plasma and sputum IL-6 and sputum IFNγ, IL-12p70, IL-1β, IL-15, and MPO. The reduction in key inflammatory signals associated with host immune defense has strong potential for pwCF [21], and the mechanisms responsible for these responses will be an exciting area for future studies. Recent preclinical studies from our laboratory using the ketone diester, R,S-1,3-butanediol diacetoacetate (KDE), provide further support for the contention that exogenous ketones provide powerful anti-inflammatory and host-defense protection. Indeed, unpublished work from our laboratory in older (72-week) C57BL/6J mice show that the ratio of CD4+/CD8+ was higher after only 8 weeks of administration, which is consistent with improved immune function [39]. Other published animal studies from our laboratory show that, in the presence of a high-fat [30] or a high-fat–high-sugar diet [40], KDE decreased hepatic lipid content and markers of inflammation, fibrogenesis, and fibrosis. These findings are particularly relevant for pwCF who are traditionally prescribed high-fat–high-calorie diets. The capacity of exogenous ketones to decrease hepatic lipid content, and hypothetically, pancreatic lipid content would be expected to improve metabolic function in pwCF. Because there is emerging evidence that the microbiome may influence pulmonary function in pwCF [41,42,43], it may also be worthwhile to examine the role ketones have and whether this might provide another mechanism to explain benefits.

## 5. Conclusions

With *CFTR* modulator therapies, there has been an increase in the prevalence of overweight and obesity in pwCF [44,45,46]. KME may serve to mitigate excess weight gain among pwCF prescribed *CFTR* modulators. Although changes in body weight were not an outcome in this study, in participants that had body weight measurements performed prior to and at discharge, we did observe a noticeable decrease in body weight in the KME group compared to PC that merit further investigation.

While the small sample size did not allow us to rigorously examine the effects of KME on lung function, inflammatory markers, and quality of life, the established benefits of nutritional ketosis on host defense, weight management, body composition, and metabolism appear to justify future studies designed to examine the potential of exogenous ketones in pwCF. *CFTR* modulation is a revolutionary treatment for CF, but the extension of lifespan will undoubtedly lead to an inflection point in clinical care, where the development of chronic diseases that were previously not experienced must be considered in contemporary CF care guidelines. Whether the use of exogenous ketones may attenuate such diseases remains to be determined. Another primary concern that needs to be addressed is the dietary recommendations for pwCF. In the past, patients were largely advised to consume high-fat and high-sugar diets in an attempt to maintain body weight. With the advent of modulator therapy, nutrition status has improved dramatically, but it is becoming increasingly clear that the development of optimal dietary regimens focused on the promotion of nutrient-dense foods that incorporate unsaturated fats, fruits, vegetables, and limited added sugar will be important [47] to attenuate a pattern of increased adiposity and inflammation. It also seems likely that variances in genotype, individual preferences and responses, and environment will all require attention to optimize precision in nutrition prescription.

## Figures and Tables

**Table 1:** Participant Characteristics.

	Overall	CON	KME	*p*-Value
Patient Status				
Inpatient	6 (42.9)	2 (40.0)	4 (44.4)	0.9999
Outpatient	6 (57.1)	3 (60.0)	5 (55.6)	
Age (y)	34.0 (21.0, 52.0)	41.0 (24.0, 52.0)	34.0 (21.0, 52.0)	0.3661
Height (in)	63.0 (54.0, 74.0)	63.0 (60.0, 72.0)	63.0 (54.0, 74.0)	0.9476
Weight (kg)	60.5 (38.5, 93.0)	61.2 (51.2, 93.0)	59.9 (38.5, 86.2)	0.9999
Sex				
Female	8 (57.1)	4 (80.1)	4 (44.4)	0.3007
Male	6 (42.1)	1 (20.0)	5 (55.6)	
Race				
African American	1 (7.1)	1 (20.0)	0 (0.0)	0.3571
European American	13 (92.9)	4 (80.0)	9 (100.0)	
CFRD				
Yes	7 (50.0)	3 (60.0)	4 (44.4)	0.9999
No	7 (50.0)	2 (40.0)	5 (55.6)	
Insulin				
Yes	6 (42.9)	2 (40.0)	4 (44.4)	0.9999
No	8 (57.1)	3 (60.0)	5 (55.6)	
Creon				
Yes	13 (92.9)	4 (80.0)	9 (100.0)	0.3571
No	1 (7.1)	1 (20.0)	0 (0.0)	
Trikafta				
Yes	14 (100.0)	5 (100.0)	9 (100.0)	-
No	0 (0.0)	0 (0.0)	0 (0.0)	

Values are total or group [control (CON) or ketone monoester (KME)] N and percent. CFRD = Cystic fibrosis-related diabetes. Patient status, sex, race, CFRD, insulin and Creon *p*-values obtained using Fisher’s exact test. Age, height, and weight *p*-values obtained using Wilcoxan-Rank Sum, t-approximation.

**Table 2:** Genotypic frequency.

Mutation A1	Mutation A2	Total n	CON	KME
F508del	F508del	5	XX	XXX
F508del	r553x	1		X
F508del	Unidentified	1	X	
F508del	q493x	1		X
F508del	s549n	1	X	
F508del	g551d	1		X
F508del	1154insTC	1		X
F508del	1558s	1		X
F508del	4077_4080delinsAA	1	X	
F508del	2183delAA	1		X

X = the frequency distribution between groups (CON vs. KME) for each allele in the study popoulation.

**Table 3:** Participant symptoms (N = 14 *).

Variable		Level	Overall	Control (N = 5)	KME (N = 9)	*p*-Value
*Day 1*	General health ^1^	Very good	1 (7.7)	0 (0.0)	1 (12.5)	--
		Good	6 (46.2)	3 (60.0)	3 (37.5)	
		Fair	5 (38.5)	2 (40.0)	3 (37.5)	
		Poor	1 (7.7)	0 (0.0)	1 (12.5)	
	Nausea	Absent	11 (78.6)	4 (80.0)	7 (77.8)	>0.9999
		Mild	1 (7.1)	0 (0.0)	1 (11.1)	
		Moderate	1 (7.1)	1 (20.0)	0 (0.0)	
		Severe	1 (7.1)	0 (0.0)	1 (11.1)	
	Vomiting	Absent	13 (92.9)	5 (100.0)	8 (88.9)	>0.9999
		Moderate	1 (7.1)	0 (0)	1 (11.1)	
	Heartburn	Absent	11 (78.6)	4 (80.0)	7 (77.8)	>0.9999
		Mild	3 (21.4)	1 (20.0)	2 (22.2)	
	Stomach pain	Absent	12 (85.7)	5 (100.0)	7 (77.8)	0.5055
		Mild	1 (7.1)	0 (0)	1 (11.1)	
		Severe	1 (7.1)	0 (0)	1 (11.1)	
	Diarrhea	Absent	11 (78.6)	4 (80.0)	7 (77.8)	>0.9999
		Mild	3 (21.4)	1 (20.0)	2 (22.2)	
	Constipation	Absent	13 (92.9)	4 (80.0)	9 (100.0)	0.3571
		Mild	1 (7.1)	1 (20.0)	0 (0)	
	Bloating	Absent	8 (57.1)	3 (60.0)	5 (55.6)	>0.9999
		Mild	4 (28.6)	1 (20.0)	3 (33.3)	
		Moderate	2 (14.3)	1 (20.0)	1 (11.1)	
	Belching	Absent	9 (64.3)	3 (60.0)	6 (66.7)	>0.9999
		Mild	5 (35.7)	2 (40.0)	3 (33.3)	
*Day 7*	General health ^1^	Very good	2 (14.3)	0 (0.0)	2 (22.2)	----
		Good	6 (42.9)	2 (40.0)	4 (44.4)	
		Fair	6 (42.9)	3 (60.0)	3 (33.3)	
	Nausea	Absent	9 (64.3)	2 (40.0)	7 (77.8)	>0.9999
		Mild	2 (14.3)	1 (20.0)	1 (11.1)	
		Moderate	1 (7.1)	1 (20.0)	0 (0)	
		Severe	2 (14.3)	1 (20.0)	1 (11.1)	
	Vomiting	Absent	14 (100.0)	5 (100.0)	9 (100.0)	NA
	Heartburn	Absent	13 (92.9)	4 (80.0)	9 (100.0)	0.3571
		Moderate	1 (7.1)	1 (20.0)	0 (0)	
	Stomach pain	Absent Mild	12 (85.7) 2 (14.3)	5 (100.0) 0 (0)	7 (77.8) 2 (22.2)	0.5055
	Diarrhea	Absent	13 (92.9)	4 (80.0)	9 (100.0)	0.3571
		Mild	1 (7.1)	1 (20.0)	0 (0.0)	
	Constipation	Absent	12 (85.7)	4 (80.0)	8 (88.9)	>0.9999
		Mild	2 (14.3)	1 (20.0)	1 (11.1)	
	Bloating	Absent	13 (92.9)	4 (80.0)	9 (100.0)	0.3571
		Mild	1 (7.1)	1 (20.0)	0 (0.0)	
	Belching	Absent	11 (78.6)	3 (60.0)	8 (88.9)	0.5055
		Mild	3 (21.4)	2 (40.0)	1 (11.1)	

* No missing data except where indicated. N = 13, 1 missing observation in KME group; ^1^ no testing performed. Fisher’s exact test; responses were dichotomized into “Present” and “Absent” for testing.

**Table 4:** CFQR Composite score, first and final days.

Variable	Overall (N = 14)		Control (N = 5)		KME (N = 9)		
*Day 1*	Mean (SD)	Median (Min, Max)	Mean (SD)	Median (Min, Max)	Mean (SD)	Median (Min, Max)	*p*-Value ^†^
Physical	57.1 (29.6)	64.6 (4.2, 95.8)	59.2 (21.9)	58.3 (37.5, 83.3)	56.0 (34.4)	70.8 (4.2, 95.8)	>0.9999
Vitality	47.0 (22.6)	45.8 (0.0, 75.0)	43.3 (19.9)	33.3 (25.0, 75.0)	49.1 (24.8)	50.0 (0.0, 75.0)	0.5518
Emotion	71.0 (25.2)	80.0 (20.0, 100.0)	73.3 (28.7)	80.0 (40.0, 100.0)	69.9 (24.7)	80.0 (20.0, 93.3)	0.7423
Eat	85.7 (21.1)	100.0 (33.3, 100.0)	80.0 (19.9)	77.8 (55.6, 100.0)	88.9 (22.2)	100.0 (33.3, 100.0)	0.3541
Treat	61.1 (25.7)	66.7 (11.1, 100.0)	55.6 (20.8)	66.7 (33.3, 77.8)	64.2 (28.7)	66.7 (11.1, 100.0)	0.5116
Health	52.4 (24.8)	55.6 (0.0, 88.9)	60.0 (16.9)	55.6 (44.4, 77.8)	48.1 (28.3)	55.6 (0.0, 88.9)	0.5120
Social	68.3 (24.8)	77.8 (5.6, 94.4)	75.6 (13.9)	83.3 (55.6, 88.9)	64.2 (29.1)	77.8 (5.6, 94.4)	0.5134
Body	62.7 (21.2)	55.6 (33.3, 100.0)	68.9 (29.8)	55.6 (33.3, 100.0)	59.3 (15.7)	55.6 (44.4, 88.9)	0.5926
Role	73.2 (24.1)	79.2 (16.7, 100.0)	73.3 (19.9)	83.3 (41.7, 91.7)	73.1 (27.3)	75.0 (16.7, 100.0)	0.9473
Weight	59.8 (39.2)	50.0 (0.0, 100.0)	73.3 (43.5)	100.0 (0.0, 100.0)	52.2 (37.1)	33.3 (3.3, 100.0)	0.4482
Respiratory	60.2 (29.5)	66.7 (3.3, 100.0)	39.6 (23.9)	50.0 (3.3, 61.1)	77.8 (26.7)	80.6 (5.6, 100.0)	0.0311 *
Digestive	80.2 (14.6)	88.9 (44.4, 100.0)	77.8 (77.8)	77.8 (66.7, 88.9)	81.5 (16.7)	88.9 (44.4, 100.0)	0.4448
*Final day*	Overall (N = 13 *)		**Control (N = 5)**		**KME (N = 8)**		
Physical	62.8 (30.0)	75.0 (4.2, 100.0)	60.0 (22.9)	58.3 (37.5, 83.3)	64.6 (35.1)	79.2 (4.2, 100.0)	0.6153
Vitality	46.2 (20.0)	41.7 (8.3, 75.0)	43.3 (19.0)	41.7 (25.0, 75.0)	47.9 (21.7)	45.8 (8.3, 75.0)	0.6113
Emotion	70.3 (25.2)	73.3 (26.7, 100.0)	72.0 (28.4)	73.3 (40.0, 100.0)	69.2 (24.9)	76.7 (26.7, 100.0)	0.9423
Eat	83.8 (22.0)	100.0 (44.4, 100.0)	75.6 (25.3)	77.8 (44.4, 100.0)	88.9 (19.7)	100.0 (44.4, 100.0)	0.3971
Treat	59.8 (29.2)	66.7 (11.1, 100.0)	48.9 (23.0)	44.4 (22.2, 77.8)	66.7 (32.0)	72.2 (11.1, 100.0)	0.3232
Health	53.0 (24.9)	55.6 (11.1, 88.9)	57.8 (12.2)	55.6 (44.4, 77.8)	50.0 (30.9)	61.1 (11.1, 88.9)	>0.9999
Social	69.3 (25.8)	77.8 (11.1, 100.0)	74.6 (13.7)	77.8 (55.6, 89.9)	66.0 (31.6)	69.4 (11.1, 100.0)	0.7691
Body	62.4 (24.7)	55.6 (33.3, 100.0)	64.4 (33.7)	55.6 (33.3, 100.0)	61.1 (19.7)	55.6 (44.4, 88.9)	>0.9999
Role	73.7 (23.8)	75.0 (25.0, 100.0)	71.7 (19.2)	75.0 (41.7, 91.7)	75.0 (27.5)	79.2 (25.0, 100.0)	0.7182
Weight	56.4 (41.7)	66.7 (0.0, 100.0)	73.3 (43.5)	100.0 (0.0, 100.0)	45.8 (39.6)	33.3 (0.0, 100.0)	0.3065
Respiratory	60.3 (26.0)	61.1 (11.1, 100.0)	44.4 (10.4)	44.4 (27.8, 55.6)	70.1 (28.5)	72.2 (11.1, 100.0)	0.0544
Digestive	79.5 (15.6)	77.8 (44.4, 100.0)	77.8 (11.1)	77.8 (66.7, 88.9)	80.6 (18.5)	83.3 (44.4, 100.0)	0.6083

* Indicates significant differences between groups (KME vs. CON). ^†^ Wilcoxon Rank-Sum, *t*-approximation, comparing values between treatment groups.

**Table 5:** Plasma Inflammatory Markers, Comparison of Treatment Groups, Median (Min, Max).

	**Placebo Control (n = 4)**		**Ketone Monoester (n = 9)**		
	**Pre**	**Post**	**Pre**	**Post**	***p*-Value**
IFNγ	3.9 (2.2, 62.3)	4.8 (3.2, 20.2)	3.8 (2.5, 35.3)	4.4 (2.7, 7.0)	0.5985
IL-10	0.5 (0.4, 3.3)	1.0 (0.4, 1.0)	0.3 (0.2, 2.3)	0.3 (0.1, 0.6)	0.3357
IL-12p70	0.4 (0.1, 0.7)	0.2 (0.2, 0.6)	0.1 (0.0, 13.7)	0.1 (0.1, 11.5)	0.2697
IL-6	1.2 (0.5, 3.0)	1.2 (0.7, 4.4)	1.0 (0.2, 3.7)	0.5 (0.2, 2.1)	0.3357
TNFα	1.8 (1.2, 4.5)	2.1 (1.4, 2.8)	1.8 (1.4, 4.3)	1.8 (1.5, 2.6)	0.4127
IL-15	2.5 (2.0, 3.6)	2.4 (1.9, 2.9)	2.5 (1.8, 3.6)	2.2 (1.8, 3.0)	0.9398
IL-16	248.9 (140.4, 351.4)	200.3 (141.3, 316.0)	194.2 (123.1, 495.0)	175.7 (98.8, 396.6)	>0.9999
IL-17	1.0 (0.5, 5.0)	1.1 (0.6, 2.7)	1.2 (0.5, 5.2)	1.0 (0.3, 3.8)	>0.9999
IL-18	810.5 (493.0, 1246.0)	740.0 (485.0, 1075.0)	534.0 (367.0, 1014.0)	602.0 (321.0, 758.0)	0.4127

Values are median (minimum, maximum). Difference values were created (post–pre) and the median of each group was compared using Wilcoxan Rank Sum, t-approximation. Significance was set a priori at *p* < 0.05.

**Table 6:** Sputum Inflammatory Outcomes.

	**Δ Placebo Control (n = 3)**	**Δ** **Ketone Monoester (n = 4)**	***p*-Value**
IFNγ	53.31 [(1.33)–(141.9)]	−3.59 [(−21.18)–(−2.280)]	0.057
IL-12p70	0.49 [(0.33)–(0.66)]	−0.30 [(−1.08)–(−0.10)]	0.057
IL-1β	331.30 [(282.7)–(414.3)]	−179.50 [(1121.0)–(154.9)]	0.100
IL-6	6.98 [(0.61)–(12.97)]	−5.64 [(−7.58)–(−1.32)]	0.057
TNFα	−28.80 [(24.35)–(−195.4)]	−26.85 [(−57.85)–(−11.71)]	1.000
IL-15	1.16 [(1.06)–(8.52)]	−0.58 [(−0.78)–(−0.09)]	0.057
IL-16	8.09 [(−14.45)–(−12.70)]	6.22 [(−947.2)–(138.5)]	0.857
IL-1α	123.50 [(−42.78)–(698.8)]	−90.47 [(−697.90)–(−29.15)]	0.114
NE	76.94 [(50.77)–(103.10)]	62.97 [(−360.9)–(152.5)]	1.000
MPO	37.08 [(36.02)–(38.14)]	−9.86 [(−28.45)–(−0.93)]	0.133
MMP9	1.43 [(−3.39)–(6.24)]	−2.94 [(−7.97)–(−1.34)]	0.533

Values are median differences (pre–post) [(min)–(max)]. Group comparisons were made using Wilcoxan Rank-Sum, t-approximation. Significance was set a priori at *p* < 0.05.

## Data Availability

Data supporting reported results can be made available.
